# Linear magnetoelectric effect in göthite, α-FeOOH

**DOI:** 10.1038/s41598-017-16772-w

**Published:** 2017-11-27

**Authors:** N. V. Ter-Oganessian, A. A. Guda, V. P. Sakhnenko

**Affiliations:** 1 Institute of Physics, Southern Federal University, Rostov-on-Don, 344090 Russian Federation; 2 International Research Center ”Smart Materials”, Southern Federal University, Rostov-on-Don, 344090 Russia

## Abstract

By means of symmetry analysis, density functional theory calculations, and Monte Carlo simulations we show that goethite, α-FeOOH, is a linear magnetoelectric below its Néel temperature *T*_N_ = 400 K. The experimentally observed magnetic field induced spin-flop phase transition results in either change of direction of electric polarization or its suppression. Estimated value of magnetoelectric coefficient is 0.57 *μ*C · m^−2^ · T^−1^. The abundance of goethite in nature makes it arguably the most widespread magnetoelectric material.

## Introduction

The field of multiferroics has become one of the focal points in condensed matter physics during the last two decades. Mutual influence of magnetic and electric subsystems in magnetoelectrics opens up new opportunities for practical applications such as, for example, new types of logical elements, devices for storage of information, and various sensors^1,2^. This stimulates search for new multiferroic materials both in the single-phase forms and as composites. Recent advances in the physics and design of magnetoelectrics were summarized in numerous reviews^3,4^.

Magnetoelectrics are known since late 1950’s and were intensively studied during the last two decades. By now, many magnetoelectric (ME) crystals or even whole classes of such compounds are identified. However, the quest for new compounds continues due to the need for higher ME coupling constants and higher working temperatures.

Iron forms many oxides and hydroxides showing a plethora of magnetic properties, which also often develop at high temperatures^5^. However, in contrast to, for example, chromium (Cr_2_O_3_)^6^, cupric (CuO)^7^, or cobalt (Co_3_O_4_)^8^ oxides, only Fe_3_O_4_ and ε-Fe_2_O_3_ were shown to display magnetoelectric properties^9–11^.

Goethite, *α*-FeOOH, is one of the most thermodynamically stable compounds out of iron oxides, hydroxides, or oxides-hydroxides, which arguably makes it the most abundant in nature among them^5^. It is found in rocks and soils and is often responsible for their colour. In many parts of the world current climate favours mineralogical transformation of hematite (*α*-Fe_2_O_3_) to goethite in soils and, therefore, the hematite-goethite ratio reflects the climate^12^. Goethite is also a common component of rusts, both atmospheric and electrochemical^5^, and is found on Mars among other iron-containing minerals^13^. In practical use goethite is an important pigment as it is a component of ochre deposits, however it also attracts interest in the form of suspensions of nanoparticles or nanorods showing considerable magnetic field-induced birefringence^14,15^.

Here we show that goethite is linear magnetoelectric below its Néel temperature *T*_N_ = 400 K making it (i) a room temperature ME material, and (ii) arguably the most abundant ME material known to date. Using density functional theory (DFT) we identify the main exchange coupling constants of goethite and confirm its antiferromagnetic ground state, whereas Monte Carlo studies uncover its magnetoelectric behavior in magnetic fields.

## Results

**Figure 1:**
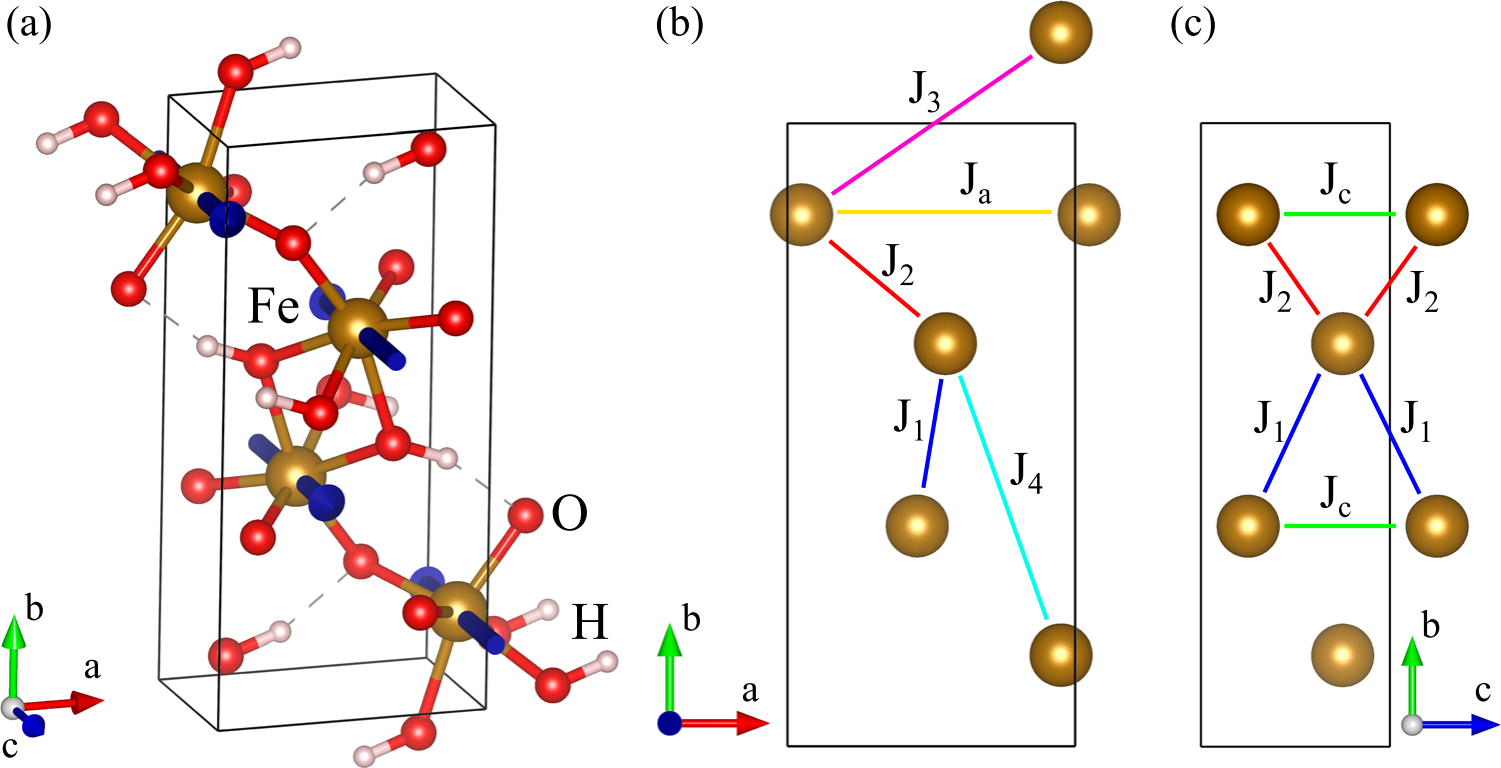
(**a**) Crystal and magnetic structures of *α*-FeOOH (blue arrows represent spins) and (**b**,**c**) magnetic exchange paths.

Goethite, *α*-FeOOH, crystallizes in the orthorhombic structure with space group symmetry Pbnm (Z = 4) shown in Fig. 1(a) and lattice parameters *a* = 4.55979 Å, *b* = 9.951 Å, and *c* = 3.0178 Å^16^. Upon decreasing temperature it experiences an antiferromagnetic phase transition at temperature *T*_N_, which varies in the range from approximately 340 to 400 K depending on the purity of the sample^17–19^. Below *T*_N_ the spins ${\overrightarrow{S}}_{i}$ of four iron ions Fe_*i*_ (*i* = 1, 2, 3, 4) located at positions (0.0489, 0.8537, 1/4), (0.9511, 0.1463, 3/4), (0.5489, 0.6463, 3/4), and (0.4511, 0.3537, 1/4)^16^, order antiferromagnetically with relative spin arrangement (+ − − +), respectively, as shown in Fig. 1(a) ^20,21^. This ordered spin arrangement can be described by the order parameter $\overrightarrow{A}$. Other possible spin arrangements with $\overrightarrow{k}=0$ described by the order parameters $\overrightarrow{F}$, $\overrightarrow{G}$, and $\overrightarrow{C}$ are summarized in Table 1. The direction of the ordered spins is experimentally found to be along the *c* axis of the crystal cell. Therefore, the appearing magnetic structure with the wave vector $\overrightarrow{k}=0$ can be described by the order parameter *A*_*z*_. Below we adopt an orthogonal system of axes *x*, *y*, and *z* being parallel to the crystal axes *a*, *b*, and *c*, respectively.

**Table 1:** Spin arrangements of the Fe_*i*_ ions with $\overrightarrow{k}=0$. First four columns give relative spin orderings of Fe_*i*_ spins. The last column gives the irreducible representations (IR) according to which the components *x*, *y*, and *z* of the order parameters transform, respectively.

Fe_1_	Fe_2_	Fe_3_	Fe_4_	Order parameter	IR’s
+	+	+	+	$\overrightarrow{F}$	Γ^2+^, Γ^3+^, Γ^4+^
+	−	+	−	$\overrightarrow{G}$	Γ^1−^, Γ^4−^, Γ^3−^
+	+	−	−	$\overrightarrow{C}$	Γ^3+^, Γ^2+^, Γ^1+^
+	−	−	+	$\overrightarrow{A}$	Γ^4−^, Γ^1−^, Γ^2−^

The symmetry of magnetic structure with A_*z*_ ≠ 0 appearing below T_*N*_ is Pb′nm^21^ and allows linear magnetoelectric effect with magnetoelectric interactions given by

1 $${A}_{z}{F}_{y}{P}_{z},$$

2 $${A}_{z}{F}_{z}{P}_{y},$$

where $\overrightarrow{F}$ and $\overrightarrow{P}$ are ferromagnetic moment and electric polarization, respectively. Thus, in the antiferromagnetic phase magnetic field applied along the *y* or *z* axis induces electric polarization components *P*_*z*_ or *P*_*y*_, respectively. It is found, however, that sufficiently strong magnetic field along the *z* axis results in a spin-flop transition, in which the spins reorient towards either the *x* or the *y* axis^18^. This will be discussed in more detail below.

It has to be noted here, that in the case when the initial paraelectric and paramagnetic phase possesses inversion symmetry operation, a magnetic phase transition with $\overrightarrow{k}=0$ occurring according to a single irreducible representation cannot induce electric polarization^22^. However, linear magnetoelectric effect can be possible, as is the case in *α*-FeOOH: when *A*_*z*_ ≠ 0 appears, the inversion symmetry is broken, but spatial inversion together with time reversal operation is a symmetry element, which results in interactions (1) and (2).

Using density functional theory we calculate six magnetic exchange constants, which are summarized in Table 2 and the respective exchange paths are shown in Fig. 1(b,c). It is found that the exchange couplings are mostly antiferromagnetic and the magnetic ground state is described by $\overrightarrow{A}\ne 0$ in accordance with the experiments.

**Table 2:** Calculated magnetic exchange constants for α-FeOOH in meV.

	J_1_	J_2_	J_3_	J_4_	J_*a*_	J_*c*_
Fe–Fe distance, Å	3.310	3.438	5.288	5.308	4.598	3.018
J, meV	15.1	48.1	−0.32	3.18	4.38	17.7

**Figure 2:**
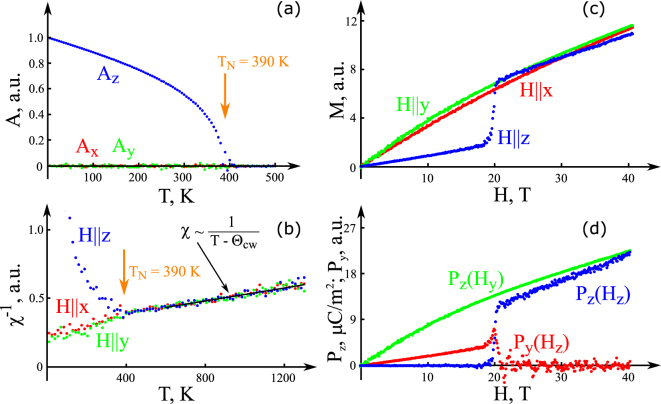
Results of Monte Carlo calculations. (**a**) Temperature dependence of the order parameters *A*_*x*_, *A*_*y*_, and *A*_*z*_. (**b**) Reciprocal magnetic susceptibility for various directions as function of temperature and a fit with the Curie-Weiss law (solid line). (**c**) Magnetization and (**d**) electric polarization at *T* = 100 K as function of magnetic field.

Monte Carlo (MC) calculations reveal that with the found exchange constants the Néel temperature ${T}_{{\rm{N}}}^{{\rm{M}}C}=390$ K is slightly lower than in experiments. Figure 2(a) shows temperature dependence of the order parameters, revealing that *A*_*z*_ emerges at ${T}_{{\rm{N}}}^{{\rm{M}}C}$ confirming the appearance of antiferromagnetic order. The fit of magnetic susceptibility in the paramagnetic region by $\chi =C/(T-{{\rm{\Theta }}}_{{\rm{C}}W})$ shown in Fig. 2(b) gives the Curie-Weiss temperature ${{\rm{\Theta }}}_{{\rm{C}}W}\approx -1250$ K. This implies that in goethite considerable magnetic frustration exists since $|{{\rm{\Theta }}}_{{\rm{CW}}}|/{T}_{{\rm{N}}}\approx 3.2$. The origin of frustration is in the presence of triangular arrangements of spins, which interact via the three dominant antiferromagnetic exchange couplings *J*_1_, *J*_2_, and *J*_c_, as shown in Fig. 1(c).

At *H*_*c*_ = 20 T a spin-flop transition occurs in goethite^18^ resulting in rotation of the antiferromagnetic vector to either *a*- or *b*-axis. The experimental value of the spin-flop magnetic field *H*_*c*_ is used to scale our results on magnetic field dependence of magnetization in the antiferromagnetic phase shown in Fig. 2(c), which are in qualitative agreement with the experimental data^18^. In our Monte Carlo simulations we assume *D*_*x*_ > *D*_*y*_, which results in appearance of *A*_*y*_ at *H*_*z*_ ≥ *H*_*c*_ and corresponding vanishing of *A*_*z*_.

Figure 2(d) shows *H*-dependence of electric polarization calculated using Eqs (1 and 2) and the ME interaction

3 $${A}_{y}{F}_{z}{P}_{z},$$

which is relevant in the spin-flopped phase in the case when *D*_*x*_ > *D*_*y*_. (As follows from Eqs (1–3) the components of electric polarization can be calculated multiplying the components of $\overrightarrow{A}$ and $\overrightarrow{F}$ obtained by Monte Carlo simulations). It follows that in the antiferromagnetic phase *α*-FeOOH is a linear magnetoelectric, since external *H*_*y*_ and *H*_*z*_ induce *P*_*z*_ and *P*_*y*_, respectively. Furthermore, at *H*_*z*_ = 20 T a flop of polarization from the *b*- to *c*-axis may occur. In the case *D*_*x*_< *D*_*y*_ the antiferromagnetic vector will flip to *A*_*x*_ at *H*_*z*_ ≥ 20 T resulting in disappearance of electric polarization.

The microscopic origin of ME effect can be understood rewriting the ME interaction (1) through spins

4 $${I}_{1}={A}_{z}{F}_{y}{P}_{z}={w}_{1}+{w}_{2}+{w}_{3}-{w}_{4},$$

where

5 $${w}_{1}={P}_{z}({S}_{1y}{S}_{1z}-{S}_{2y}{S}_{2z}-{S}_{3y}{S}_{3z}+{S}_{4y}{S}_{4z}),$$

6 $${w}_{2}={P}_{z}({S}_{1z}{S}_{2y}-{S}_{1y}{S}_{2z}-{S}_{3z}{S}_{4y}+{S}_{3y}{S}_{4z}),$$

7 $${w}_{3}={P}_{z}({S}_{1z}{S}_{3y}-{S}_{1y}{S}_{3z}-{S}_{2z}{S}_{4y}+{S}_{2y}{S}_{4z}),$$

8 $${w}_{4}={P}_{z}({S}_{2z}{S}_{3y}+{S}_{2y}{S}_{3z}-{S}_{1z}{S}_{4y}-{S}_{1y}{S}_{4z}).$$

The interaction *w*_1_ is a single-ion contribution, whereas *w*_2_, *w*_3_, and *w*_4_ result from interactions of two spins. Thus, the ME coupling may have both single-ion and two-ion contributions. The single-ion contribution is in accordance with the local non-centrosymmetric crystal environment of Fe atoms, the local crystal symmetry of which is a mirror plane σ_*z*_ oriented parallel to the *xy* plane. Thus, it allows local spin-dependent electric dipole moments of electron orbitals *d*_*z*_ ~ *S*_*y*_ *S*_*z*_^23^.

In order to estimate the value of magnetically induced electric polarization we performed non-collinear DFT calculations. The spins were first relaxed in the stable *A*_*z*_ configuration and then constrained to give additional ferromagnetic component *F*_*y*_. Artificially induced ferromagnetic ordering amounted to approximately 0.63 *μ*_B_ per spin (while the antiferromagnetic component *A*_*z*_ to approximately 4.09 *μ*_B_ per spin), which resulted in rotation of spins away from the *z* axis by about 8.8°. The resulting electric polarization calculated using the Berry phase approach was found to take the value of 120 *μ*C/m^2^. Taking the experimental magnetic susceptibility of approximately 0.003 *μ*_B_/T per Fe^3+^ ion^24,25^ we can estimate the ME coefficient to be of the order of 0.57 *μ*C · m^−2^ · T^−1^, which is comparable to that of LiNiPO_4_^23,26^.

When *D*_*x*_ > *D*_*y*_ and the external magnetic field is higher than the spin-flop field *H*_*z*_ > *H*_*c*_, the antiferromagnetic vector changes to *A*_*y*_ ≠ 0 and *P*_*z*_ appears. DFT calculations in this phase give value of the ME coefficient *∂P*_*z*_/*∂H*_*z*_ very close to the value of *∂P*_*z*_/*∂H*_*y*_ in the phase *A*_*z*_ ≠ 0 calculated above. These ME coefficients together with the spin-flop field value allow to set scaling of *P*_*z*_ and magnetic field in Fig. 2(d). However, the ME coefficient *∂P*_*y*_/*∂H*_*z*_ in the low field phase *A*_*z*_ ≠ 0 depends on the unknown magnetic susceptibility ${\chi }_{\parallel }={\rm{\partial }}{M}_{z}/{\rm{\partial }}{H}_{z}$, which should be much lower, though, than χ_⊥_ = ∂*M*_*y*_/∂*H*_*y*_, precluding from setting reliable scale for *P*_*y*_ in Fig. 2(d).

Relative values of different contributions to ME effect can be estimated from DFT calculations. For this purpose one can use the ME interactions

9 $${I}_{2}={C}_{z}{G}_{y}{P}_{z}={w}_{1}-{w}_{2}+{w}_{3}+{w}_{4},$$

10 $${I}_{3}={A}_{y}{F}_{z}{P}_{z}={w}_{1}-{w}_{2}-{w}_{3}-{w}_{4},$$

11 $${I}_{4}={C}_{y}{G}_{z}{P}_{z}={w}_{1}+{w}_{2}-{w}_{3}+{w}_{4}.$$

Performing calculations using the magnetic configurations *C*_*z*_ *C*_*y*_, *A*_*y*_ *F*_*z*_, and *C*_*z*_ *C*_*y*_ similar to above and evaluating *P*_*z*_ using the Berry phase approach we find that the biggest contribution to ME effect is *w*_3_ and the other contributions relative to *w*_3_ are *w*_1_/*w*_3_ ≈ −0.034, *w*_2_/*w*_3_ ≈ −0.21, and *w*_4_/*w*_3_ = 0. Therefore, it follows that *w*_1_ and *w*_2_ act in the direction opposite to *w*_3_.

## Conclusions

Based on the symmetry analysis of the available crystal and magnetic structures of goethite, *α*-FeOOH, we suggest that it is linear magnetoelectric below its Néel temperature. Using density functional calculations and Monte Carlo simulations we find main exchange constants in goethite and calculate its magnetic and magnetoelectric behavior.

Goethite belongs to the *α*-AlOOH diaspore structural type, which is also shared by, for example, *α*-MnOOH, Fe(OH)F, and Co(OH)F. The latter compound is also antiferromagnetic below ~40 K with the spin arrangement similar to *α*-FeOOH^27^ and should, thus, display similar linear ME properties below its *T*_*N*_.

Nature creates beautiful polycrystalline goethite samples, which are encountered in significant amounts in various deposits. However, synthesis of single crystals in laboratory or preparation of good ceramic samples can be a challenge, as *α*-FeOOH starts to decompose at temperatures higher than 200 °C to form hematite, *α*-Fe_2_O_3_ In this respect it may be easier to show the magnetoelectric behavior experimentally in the aforementioned isostructural compounds with similar magnetic structure, e.g., in Co(OH)F.

## Methods

### DFT calculations

Density functional theory calculations were performed using the Vienna *Ab-initio* Simulation Package (VASP)^28^ and the projected augmented wave method^29^. We used the GGA exchange correlation approximation corrected by means of the GGA + U formalism for the Fe atoms with *U*_*eff*_ = *U* − *J* = 3 eV within the Dudarev approach^30^. This value of *U*_*eff*_ was shown earlier to properly account for the structural and magnetic properties of *α*-FeOOH^31,32^. The energy cutoff was 850 eV, whereas the Brillouin zone integration was done using the 8 × 4 × 12 set of *k*-points determined by the Monkhorst-Pack scheme, which provided both the energy and k-points convergence.

Spin polarized collinear calculations were used to determine the exchange constants. For the determination of 6 exchange couplings the Hamiltonian was fitted to relative total energies of 7 different collinear magnetic structures. Magnetic cell sizes included *a* × *b* × *c*, 2*a* × *b* × *c*, *a* × *b* × 2*c*, and 2*a* × 2*b* × *c*, with the corresponding changes in the *k*-points grid. The crystal structure was relaxed in the most stable $\overrightarrow{A}$ magnetic structure using the stopping criterion for absolute values of forces on atoms of 10^−3^ eV/Å, while fixing the atomic positions for total energy calculations of other magnetic structures.

Electric polarization was calculated using the Berry phase approach as implemented in VASP while including spin-orbit coupling and performing fully non-collinear magnetic calculations. Since electric polarization appears in non-collinear magnetic structures the directions of local magnetic moments were constrained to form slightly non-collinear magnetic structure, while allowing for atomic and structural relaxation.

The calculated lattice parameters *a* = 4.638 Å, *b* = 10.037 Å, and *c* = 3.038 Å are within 1% of the experimentally determined values^16,21^. The local magnetic moment value of 4.14 *μ*_B_ of Fe ions is between the experimentally reported values of 3.8 *μ*_B_^33^ and 4.45 *μ*_B_^21^. The band gap 1.9 eV obtained in DFT calculations is slightly lower than the experimental values 2.1–2.5 eV^34,35^.

### Classical Monte-Carlo simulations

Classical Monte Carlo simulations using the exchange constants determined by DFT calculations are performed using the Hamiltonian

12 $$ {\mathcal H} =\sum _{ij}{J}_{ij}{\overrightarrow{S}}_{i}\cdot {\overrightarrow{S}}_{j}+\sum _{i}({D}_{x}{S}_{ix}^{2}+{D}_{y}{S}_{iy}^{2}+{D}_{z}{S}_{iz}^{2})-\overrightarrow{H}\cdot \overrightarrow{S},$$

where $\overrightarrow{S}$ are classical vectors of unit length, *D*_*α*_ (*α* = *x*, *y*, *z*) are anisotropy constants, and $\overrightarrow{H}$ is magnetic field. In the Hamiltonian (12) we account only for the anisotropy terms *D*_*α*_ ≠ 0 pursuing a minimal model, which reproduces the ground state *A*_*z*_ ≠ 0. However, it has to be noted that there exist other anisotropy terms, which can generate some *G*-type contribution to the magnetic structure in the spin-flopped phase (i.e., when *A*_*x*_ ≠ 0 or *A*_*y*_ ≠ 0) when magnetic field is applied along the z direction, but not in the ground state, since *A*_*z*_ is the only order parameter transforming according to the irreducible representation Γ^2−^. We assume that such interactions are small and will only slightly modify the results quantitatively. In our simulations we tentatively use *D*_*x*_ = −*D*_*z*_ = 1.5 meV and *D*_*y*_ = 0, which reflects the easy axis direction parallel to the *c*-axis.

The calculations are performed using the Metropolis scheme and a simulation box with dimensions 12 × 12 × 12 unit cells. After every change in temperature or external magnetic field the system is allowed to relax for 5·10^3^ Monte Carlo steps per spin (MCS), whereas the statistical information is subsequently gathered over the next 15·10^3^ MCS. Simulations of larger systems with dimensions 18 × 18 × 18 and 24 × 24 × 24 resulted in only slight increase of *T*_*N*_ by approximately 2% and 3%, respectively.
